# Continuous Influenza Virus Production in Cell Culture Shows a Periodic Accumulation of Defective Interfering Particles

**DOI:** 10.1371/journal.pone.0072288

**Published:** 2013-09-05

**Authors:** Timo Frensing, Frank Stefan Heldt, Antje Pflugmacher, Ilona Behrendt, Ingo Jordan, Dietrich Flockerzi, Yvonne Genzel, Udo Reichl

**Affiliations:** 1 Bioprocess Engineering, Max Planck Institute for Dynamics of Complex Technical Systems, Magdeburg, Germany; 2 ProBioGen AG, Berlin, Germany; 3 Systems and Control Theory, Max Planck Institute for Dynamics of Complex Technical Systems, Magdeburg, Germany; 4 Chair for Bioprocess Engineering, Otto-von-Guericke-University Magdeburg, Magdeburg, Germany; German Primate Center, Germany

## Abstract

Influenza viruses are a major public health burden during seasonal epidemics and a continuous threat due to their potential to cause pandemics. Annual vaccination provides the best protection against the contagious respiratory illness caused by influenza viruses. However, the current production capacities for influenza vaccines are insufficient to meet the increasing demands. We explored the possibility to establish a continuous production process for influenza viruses using the duck-derived suspension cell line AGE1.CR. A two-stage bioreactor setup was designed in which cells were cultivated in a first stirred tank reactor where an almost constant cell concentration was maintained. Cells were then constantly fed to a second bioreactor where virus infection and replication took place. Using this two-stage reactor system, it was possible to continuously produce influenza viruses. Surprisingly, virus titers showed a periodic increase and decrease during the run-time of 17 days. These titer fluctuations were caused by the presence of defective interfering particles (DIPs), which we detected by PCR. Mathematical modeling confirmed this observation showing that constant virus titers can only emerge in the absence of DIPs. Even with very low amounts of DIPs in the seed virus and very low rates for *de novo* DIP generation, defective viruses rapidly accumulate and, therefore, represent a serious challenge for continuous vaccine production. Yet, the continuous replication of influenza virus using a two-stage bioreactor setup is a novel tool to study aspects of viral evolution and the impact of DIPs.

## Introduction

Influenza viruses belong to the family of *Orthomyxoviridea*. They are enveloped viruses with a segmented single-stranded RNA genome of negative polarity. The genome of influenza A viruses consists of eight genome segments encoding up to 13 polypeptides [1], [2]. Influenza viruses cause respiratory illness and represent a high burden for human health. Annually, influenza A and B viruses account for 3 to 5 million cases of severe illness and 250,000 to 500,000 deaths worldwide (estimations from the World Health Organization). Vaccination provides the best protection against influenza. The majority of vaccine doses are being produced in embryonated chicken eggs. While egg-based influenza vaccines have a proven safety and efficacy record, their manufacturing is associated with severe limitations. These limitations include complex logistics for the supply of millions of embryonated eggs, constrains in scale-up, and low yields for some strains. Cell culture-based processes with highly susceptible mammalian cell lines have become an important alternative to embryonated chicken eggs [3]. Important advantages include that the supply with continuous cell lines is essentially unlimited and unaffected by avian influenza viruses that threaten laying flocks. Moreover, in the controlled environment of a bioreactor, sterility can easier be maintained than in egg-based production [4]. Vaccine manufacturers use predominantly Madin-Darby canine kidney (MDCK) cells or African green monkey kidney cells (Vero). In addition, other substrates such as designer cell lines are under investigation [5].

The continuous avian cell line AGE1.CR was designed for the production of vaccine viruses that replicate in avian hosts [6]. For cell line development, primary cells were obtained from the retina of a muscovy duck embryo and immortalization was achieved by stable expression of the human adenovirus E1A and E1B genes. AGE1.CR cells were adapted to growth in single cell suspension in chemically defined medium [7]. Documentation for this cell line is exhaustive and complete, and the biochemical mechanisms for immortalization are known to regulatory authorities. Hence, AGE1.CR cells can be regarded as a modern vaccine production substrate. Our group tested AGE1.CR cells for the production of influenza viruses and demonstrated their suitability for the propagation of different strains including live attenuated influenza viruses [8], [9].

Typically, cell culture-based processes for the production of influenza vaccines rely on batch cultivations [5]. These are characterized by a stepwise scale-up of cells, followed by the virus infection at high cell densities in the final production volume, and the subsequent harvest which terminates the process. Continuous virus production processes could offer advantages with respect to productivity by decreasing equipment as well as facility size and by reducing process cycle times. In addition, product quality could be improved by the establishment of a steady-state operation under constant control of key parameters and by avoiding batch-to-batch variations.

Here, we explored the possibility to establish a continuous production process for influenza viruses based on a two-stage bioreactor setup. AGE1.CR cells were cultivated in a first stirred tank bioreactor (STR) such that a stable cell concentration was maintained. From this bioreactor, the suspension cells were continuously fed into a second bioreactor where virus infection and replication took place.

## Materials and Methods

### Cells and virus

The duck-derived suspension cell line AGE1.CR [6] was cultivated in chemically defined CD-U3 medium (PAA), an improved version of CD-U2, supplemented with glutamine, alanine (both 2 mM final concentration, Sigma) and recombinant insulin-like growth factor (LONG-R^3^ IGF, 10 ng/mL final concentration, Sigma).

The human influenza virus A/Puerto Rico/8/34 H1N1 (Robert Koch Institute, #3138) was adapted to the AGE1.CR cell line over several passages. The resulting AGE1.CR adapted virus seed (Tissue culture infectious dose 50 (TCID_50_)  = 6.6×10^7^ virions/mL (used for the first continuous cultivation); TCID_50_ = 1.6×10^8^ virions/mL (second run)) was stored in aliquots at −80°C.

### Two-stage bioreactor setup for continuous influenza virus propagation

**Figure 1:**
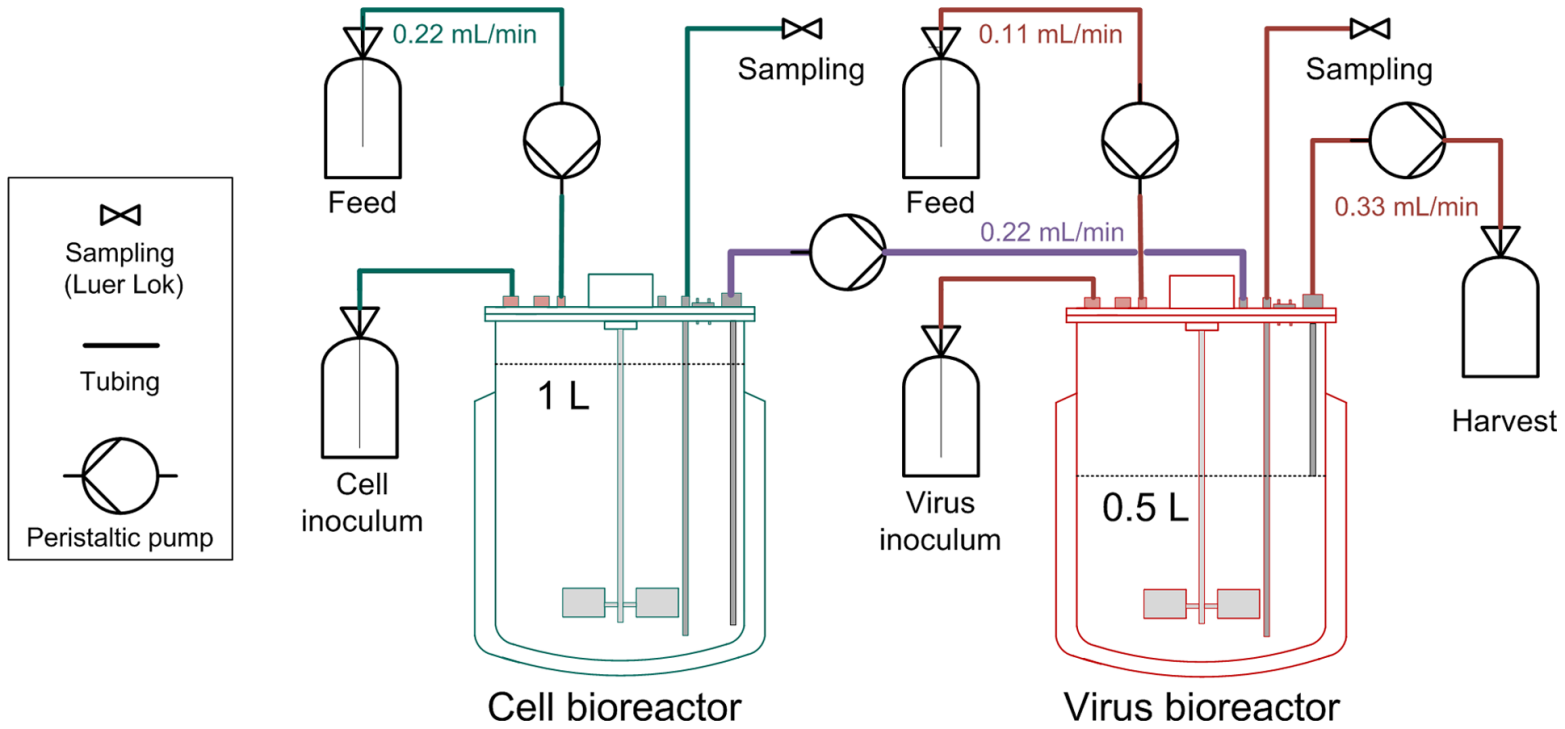
Overview of two-stage bioreactor setup for continuous virus propagation. AGE1.CR cells were cultivated in two bioreactors. At time of infection, the influenza strain A/Puerto Rico/8/34 was added to the virus bioreactor at a multiplicity of infection of 0.025. Subsequently, the cell concentration in the cell bioreactor was kept at approx. 4–5×10^6^ cells/mL and cells were constantly fed into the virus bioreactor (feeding rates are depicted). Trypsin was added either to the feed or directly into the virus bioreactor. All green components correspond to the cell bioreactor, all red components to the virus bioreactor. Both reactors are connected via the purple tubing.

Two small scale stirred tank bioreactors (1 L working volume Biostat B plus, Sartorius) were used. The first lab-scale bioreactor was inoculated with AGE1.CR cells and cultivations were carried out at 37°C, pH 7.2 and a stirring speed of 120 rpm with a working volume of 1 L. Aeration was controlled to 40% DO by pulsed aeration with pure oxygen through a microsparger. When cell concentrations reached levels of more than 4×10^6^ cells/mL, 0.375 L were transferred to the second STR. By addition of fresh medium, the working volume was adjusted to 1 L and 0.5 L in the first and second STR, respectively. Cells were cultivated for additional 24 h in batch mode. Thereafter, influenza virus was added to the second STR (virus bioreactor) at a multiplicity of infection (MOI) of 0.025 based on the viable cell count and the TCID_50_ of the seed virus. Additionally, the virus inoculum was supplemented with 1×10^−6^ U/cell trypsin (Gibco, #27250–018; sterile-filtered stock solution prepared in PBS to 500 U/mL and stored at −20°C). One hour after infection, the two STRs were operated in continuous mode with flow rates depicted in figure 1. During the first run, trypsin (3600 U/L) was present in the feed medium of the virus bioreactor. In contrast, trypsin was added directly into the virus bioreactor (5000 U once a day) in the second run to avoid self-degradation of trypsin. The feed media of both reactors were provided in 5 L bottles that were chilled and only the feed medium of the cell bioreactor had to be refilled during the run-time. Samples were taken every 12 h from both bioreactors, except for the virus reactor during the first continuous cultivation which was sampled every 6 h. From these samples, viable cell concentrations were directly determined using the trypan blue dye exclusion method automatically performed by a Vi-CELL XR instrument (Beckman Coulter). Additionally, aliquots for the TCID_50_ assay [10] were frozen at −80°C. Samples for the hemagglutination (HA) assay [11] were centrifuged for 5 min at 300 g, and supernatants were stored subsequently at −80°C. In addition, up to 0.33 mL/min of the virus reactor medium were continuously harvested to maintain 0.5 L working volume in the STR. Both runs were terminated 17 days post infection.

#### Influenza virus segment-specific PCR

To analyze viral genomes, bioreactor samples were centrifuged for 5 min at 1000 g and viral RNA was purified from 150 µL supernatant using the NucleoSpin RNA Virus extraction kit (Macherey-Nagel) according to the manufacturer's instructions.

A reverse transcription was performed by using the Uni12-primer [12]. Briefly, 10 µL of viral RNA were mixed with 1 µL primer (10 µM), 1 µL dNTPs (10 mM each) and 2.5 µL nuclease-free water. The mixture was incubated for 5 min at 65°C and subsequently cooled down to 4°C. Thereafter, a reaction mixture containing 4 µL 5× reaction buffer, 0.5 µL RevertAid H Minus M-MuLV reverse transcriptase (200 U/µL, Thermo Scientific) and 1 µL nuclease-free water was added. After incubation at 45°C for 60 min, the reaction was terminated at 70°C for 10 min.

For the segment-specific PCR eight primer pairs were used (Table 1). 2 µL of cDNA were mixed with 4 µL 5× Phusion HF buffer, 2 µL MgCL_2_ (10 mM), 1 µL dNTPs (10 mM each), 1 µL forward primer (10 µM), 1 µL reverse primer (10 µM), 0.2 µL Phusion DNA polymerase (2 U/µL) (Thermo Scientific) and 8.8 µL nuclease-free water. Initial denaturation was performed at 98°C for 3 min followed by 25 cycles with 98°C for 25 sec, 60°C (or 55°C for segment 6) for 45 sec and 72°C for 1–2 min (depending on segment length). The final elongation was performed at 72°C for 10 min. PCR products were visualized using gel electrophoresis.

**Table 1:** Primers used for the segment-specific PCR.

Primer name	Sequence 5′–3′
Seg 1 for	AGCGAAAGCAGGTCAATTAT
Seg 1 rev	AGTAGAAACAAGGTCGTTTTTAAAC
Seg 2 for	AGCGAAAGCAGGCAAACCAT
Seg 2 rev	AGTAGGAACAAGGCATTTTTTCATG
Seg 3 for	AGCGAAAGCAGGTACTGATCC
Seg 3 rev	AGTAGAAACAAGGTACTTTTTTGG
Seg 4 for	AGCAAAAGCAGGGGAA
Seg 4 rev	AGTAGAAACAAGGGTGTTTT
Seg 5 for	AGCAAAAGCAGGGTAGATAATC
Seg 5 rev	AGTAGAAACAAGGGTATTTTTC
Seg 6 for	AGCGAAAGCAGGAGT
Seg 6 rev	AGTAGAAACAAGGAGTTTTTT
Seg 7 for	AGCGAAAGCAGGTAG
Seg 7 rev	AGTAGAAACAAGGTAGTTTTT
Seg 8 for	AGAAAAAGCAGGGTGACAAA
Seg 8 rev	AGTAGAAACAAGGGTGTTTT

### Mathematical modeling

To systematically study our continuous virus production system, we developed a segregated mathematical model based on an existing description of a batch process proposed by Kirkwood and Bangham [13]. The model describes the virus reactor and comprises three key components: the number of uninfected cells, infected cells and virus particles.

**Figure 2:**
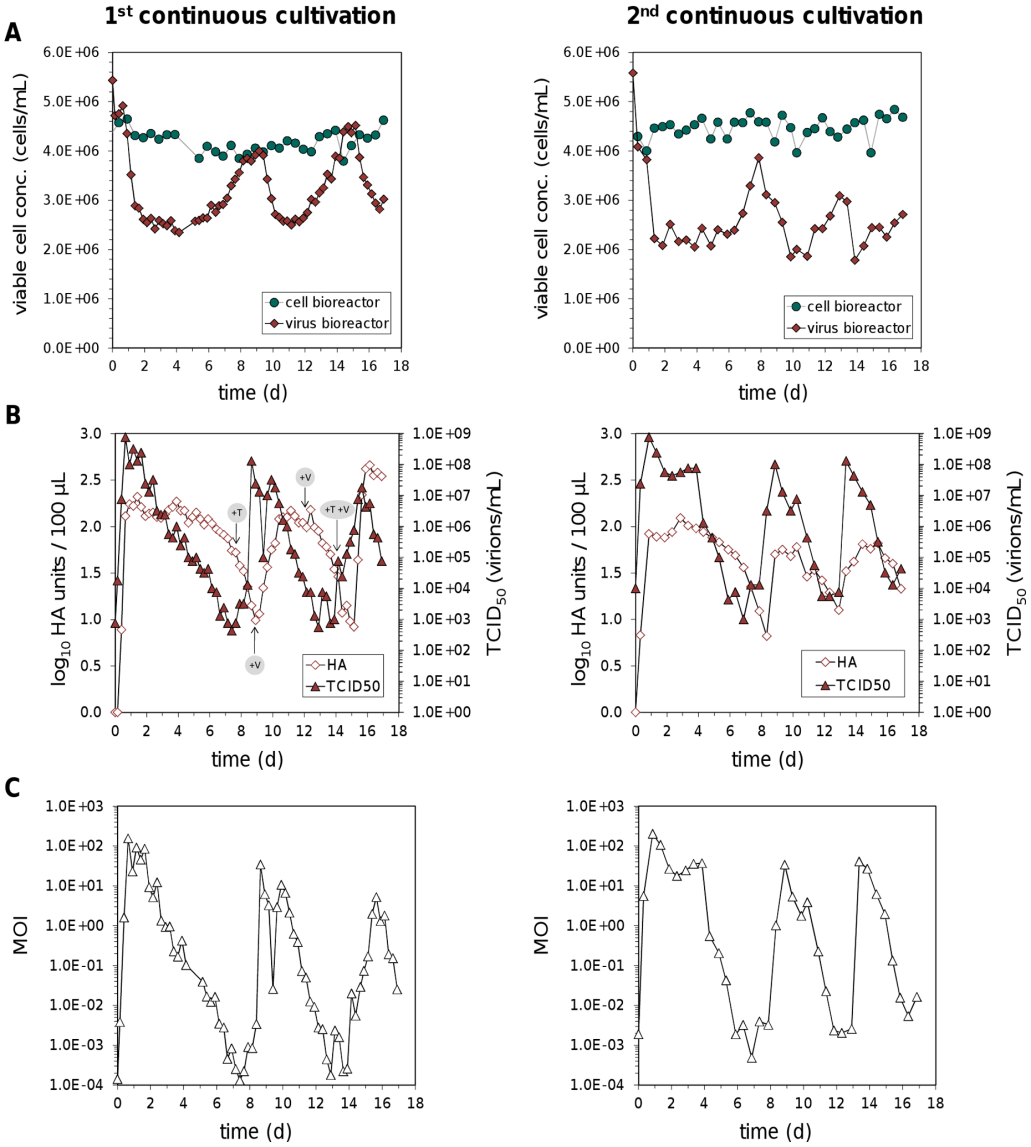
Continuous propagation of influenza A virus. (A) Concentrations of AGE1.CR cells in the cell and virus bioreactor. (B) Virus titers determined by HA and TCID_50_ assay. (C) MOI in the virus bioreactor based on the ratio of TCID_50_ to cell count at each sampling time point. Results of two independent cultivations are shown. During the first cultivation additional trypsin (+T), seed virus (+V) or both were added to the virus bioreactor at indicated time points as an attempt to counteract decreasing virus titers.

The concentration of uninfected target cells (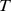) is given by
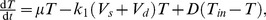
(1a)where 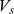 and 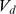 denote the concentrations of STVs and DIPs, respectively. We assume that cells grow exponentially with rate constant 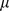 and become infected by virus particles with rate 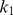, which is the same for both virus types. The last term in Eq. (1a) accounts for the continuous feed of cells with concentration 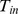 and the harvest with 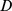 denoting the virus reactor's dilution rate. Here, ideal mixing is assumed. With respect to the roughly constant concentration of cells observed in the cell reactor (Figure 2A), we choose 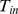 to be independent of time. In general, 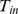 may vary in the initial phase of cultivation to a certain degree until the cell reactor reaches steady state.

The population of infected cells is subdivided into cells infected with STVs (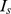) or DIPs (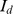) as well as co-infected cells (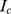)
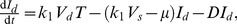
(1b)

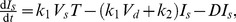
(1c)

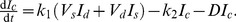
(1d)The first term in Eq. (1b) and (1c) accounts for the infection of target cells by both viruses. Similarly, infection of 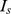 and 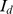 by DIPs and STVs, respectively, yields co-infected cells in Eq. (1d). Since, DIPs do not replicate in the absence of a STV, we assume that DIP-infection alone does not interfere with normal cellular processes. Hence, DIP-infected cells may continue to grow giving rise to infected daughter cells, an assumption already used by Kirkwood and Bangham [13]. Furthermore, 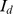 cannot revert back to the uninfected state by virus degradation. In cells infected with the STV or co-infected with both types of particles replication takes place resulting in virus-induced apoptosis with rate 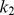. Again, the last term in Eq. (1b)–(1d) accounts for the dilution of the reactor content. To keep the model simple, we neglected that DIPs may not interfere with STV replication after it is well advanced. Kirkwood and Bangham accounted for this by introducing further subclasses of cells which track the infection age, i.e., the time that has elapsed since infection [13]. However, such subclasses severely increase the dimensionality of the model and impair a mathematical analysis.

Finally, the concentration of STVs (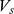) and DIPs (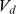) follow as
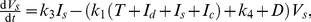
(1e)

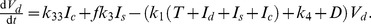
(1f)


We assume that STV-infected cells produce primarily STVs with rate 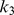 and a small fraction 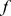 of DIPs. In contrast, co-infected cells exclusively produce DIPs with rate 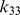. For numerical simulations we choose 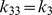. But for later analysis it is more convenient to keep a separate notation for virus production by co-infected cells. Note that free virus particles are taken up by all four cell types or degrade with rate 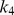.

As we will prove later, the system (1) reduces to a three dimensional model of virus growth if the seed virus is free of DIPs (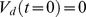), no DIP-infected or co-infected cells are present in the beginning (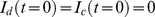) and cells infected by STVs do not generate DIPs *de novo* (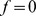)
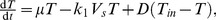
(2a)

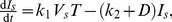
(2b)

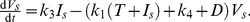
(2c)


For both the full model and the reduced version, we chose parameters and initial conditions according to Table S1 if not stated otherwise. We solved the models numerically using the CVODE routine from SUNDIALS [14] on a Linux-based system. Model files were handled with the Systems Biology Toolbox 2 [15] for MatLab® (version 7.11.0 R2010b, The MathWorks, Inc.).

## Results and Discussion

In order to establish a continuous production process for influenza viruses we used duck-derived AGE1.CR cells. Their growth in suspension is a basic prerequisite for continuous cultivations and enables an easy transfer of cells between bioreactors. In addition, for the double-stranded DNA virus MVA (modified vaccinia Ankara; highly attenuated derivative of mammalian orthopoxvirus), it has been demonstrated that AGE1.CR cells maintain permissivity and high virus yields after long-term cell cultivation with up to 318 serial passages. At this high passage level, AGE1.CR cells also preserved the expression of the introduced E1A gene [7]. Thus, the AGE1.CR cell line is suited for continuous cultivations.

A setup with two stirred tank bioreactors (STRs) was chosen (Figure 1) to establish an uninterrupted supply of cells for the spatially separated continuous virus propagation. At first in batch mode, AGE1.CR cells were propagated in one bioreactor (cell bioreactor) in a volume of 1 L until a cell concentration of more than 4×10^6^ cells/mL was reached. Then 0.375 L were transferred to the second STR. Subsequently, fresh medium was added to adjust the first STR to 1 L again and the second STR to 0.5 L working volume. One day after the transfer of cells, the influenza virus A/Puerto Rico/8/34 was added to the second STR (virus bioreactor) at a multiplicity of infection (MOI) of 0.025. The continuous culture was started by a constant feed of 0.22 mL/min of fresh medium into the cell bioreactor where the cell concentration was maintained between 3.8–4.8×10^6^ cells/mL. Cells were constantly fed from the cell bioreactor into the virus bioreactor (also at 0.22 mL/min). In addition, 0.11 mL/min of fresh medium was added to the virus reactor and up to 0.33 mL/min was harvested to maintain the 0.5 L working volume.

During the first continuous influenza virus production, cell concentrations in the cell bioreactor only fluctuated within the error range of the cell count instrument (maximum relative standard deviation 5%) (Figure 2A). In contrast, cell numbers in the virus bioreactor decreased within the first days to 2.4×10^6^ cells/mL at day 4 post infection (p.i.). This was caused by an extensive production of infectious viruses that reached concentrations of 7.6×10^8^ virions/mL already 16 h p.i. (Figure 2B). Surprisingly, cell numbers increased again between day 4 and 8 p.i. whereas virus titers decreased. Actually, the titer of infectious virus particles could not be determined during the run since the procedure of the tissue culture infectious dose 50 (TCID_50_) assay takes three days. Instead, the hemagglutination (HA) titer was monitored on a daily basis, which represents the total virus particle concentration (infectious as well as non-infectious). As HA titers decreased by day 7 p.i. while cell concentrations increased, additional trypsin was injected into the virus bioreactor. At this point, it was assumed that the trypsin supplementation of 3600 units/L in the feed medium of the virus bioreactor might not be sufficient. However, HA titers kept declining showing that trypsin activity was not limiting virus propagation. Hence, additional seed virus was injected into the virus reactor at day 9 p.i. as an attempt to restore virus propagation. Yet, the subsequent analysis of virus titers by the TCID_50_ assay revealed that infectious titers were already at high levels before we added the new seed virus which indicates that infectious virus particles were also not a limiting factor. Also at later time points, the injection of either extra seed virus alone or in combination with additional trypsin had no impact on the decrease of HA titers.

In order to establish more stable continuous virus propagation, the cultivation procedure was slightly modified in the second run. This time, instead of trypsin being added to the feed medium of the virus bioreactor, fresh trypsin was injected directly into the STR on a daily basis to avoid loss of trypsin activity due to self-degradation. Furthermore, another batch of seed virus with a higher infectious virus titer was used and no additional seed virus was added after the initial infection of the virus bioreactor (MOI 0.025). However, a similar periodic increase and decrease of virus titers was observed during this second continuous virus cultivation (Figure 2B, right panel). In general, after the initial increase of infectious titers the levels remained high for several days during both cultivations but declined by five to six orders of magnitude reaching their minimum around day 7 p.i. Thereafter, infectious titers rose again and reached a new maximum around day 9 p.i. By contrast, HA titers lagged behind infectious titers by at least one day with the first HA minimum being measured between day 8 and 9 after infection.

Based on the infectious virus titer and corresponding viable cell concentration, the MOI that occurred in the virus reactor was calculated for every sampling time point. Both cultivations were infected at an MOI of 0.025 and therefore low MOIs were obtained at the first sampling time point directly after infection and the start of continuous operation mode (Figure 2C). However, already one day after the infection, large amounts of progeny virions were released and the MOI reached levels of up to 200. The MOI values remained high for up to four days and dropped thereafter to values in the range of 10^−3^ to 10^−4^. Overall, because variations in cell concentrations were low, the dynamics of MOIs matched the course of infectious viral titers and fluctuated within six orders of magnitude.

Successive passaging of influenza virus at high MOI is known to support the accumulation of defective interfering particles (DIPs). This phenomenon was first described in the early 1950 s by von Magnus who reported the formation of what he called “incomplete” virus after undiluted serial passages in embryonated eggs [16]. In 1970, the term defective interfering particles was used to describe these particles more precisely as they are characterized by a defective genome that depends on a complete helper virus for its replication. During such a co-infection, DIPs interfere with the replication of non-defective homologous standard virus [17]. In the case of influenza virus with its segmented negative-strand RNA genome, DIPs contain one or more defective interfering (DI) RNAs that arise from internal deletions [18]. These DI RNAs predominantly originate from polymerase genes [19].

**Figure 3:**
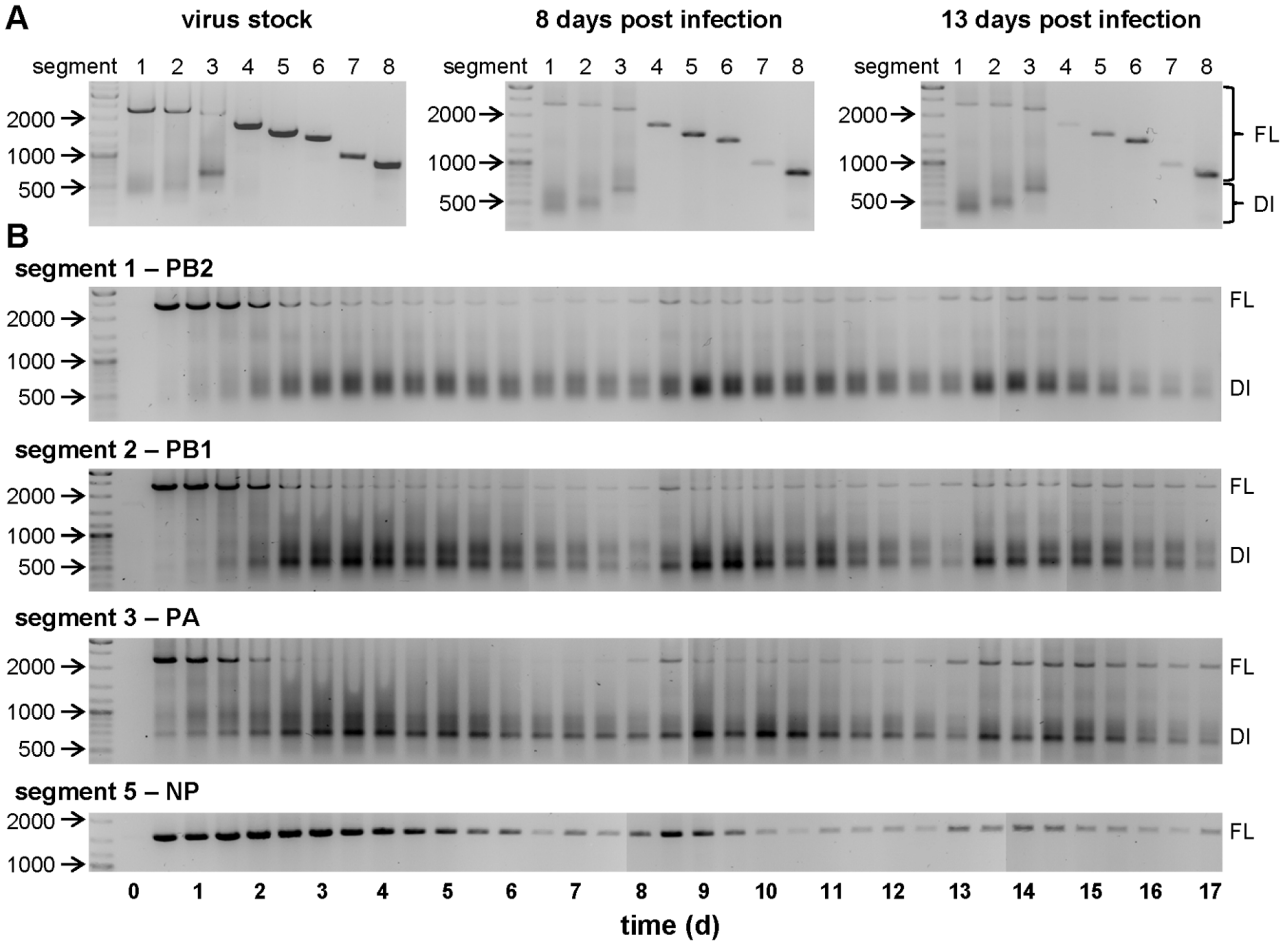
Segment-specific PCR for the detection of full-length and defective interfering genome segments. Using eight primer pairs directed against the 5′ and 3′-end of each influenza virus genome segment, full-length (FL) as well as defective interfering (DI) RNAs (smaller products in the range between 500 and 700 bp) were amplified. (A) Segment-specific PCR for all eight segments of the used virus stock and two samples of the second continuous cultivation. (B) Time course of the three polymerase segments 1–3 (encoding PB2, PB1 and PA, respectively) and segment 5 encoding the nucleoprotein (NP) from the second continuous virus propagation in which samples were taken every 12 hours. The size (in bp) of important marker bands is shown.

To analyze whether DIPs were causing fluctuations in viral titers during continuous cultivations, we looked for defective interfering RNAs in virus samples using a PCR. Therefore, cell culture samples were centrifuged for 5 min at 1000 g and 150 µL of the supernatant was used for viral RNA purification. Subsequently equal volumes of each sample were used for the reverse transcription and for the PCR reaction. Since DI RNAs arise from internal deletions and accordingly contain both the 5′ and 3′ termini of a gene segment, primers binding to those ends are able to amplify full-length as well as DI RNAs. A segment-specific PCR revealed that the used virus stock contained all eight genome segments as expected. However, for the polymerase segments 1–3 encoding PB1, PB2 and PA additional smaller PCR products in the range between 500 to 700 base pairs (bp) could be detected as faint bands or as less distinct smear (Figure 3A). In addition, two samples (8 and 13 d p.i.) from the second continuous cultivation also contained these DI RNAs while the amount of full-length segments seemed to be reduced. Hence, we took a closer look at the replication dynamics of full-length and DI RNAs during the second continuous cultivation by analyzing all cell culture supernatants sampled within the run-time of 17 days. For this study PCRs were performed for the three polymerase genes that showed DI RNAs previously, as well as for segment 5 that encodes the nucleoprotein (NP) and is one of the segments not prone to DI RNA formation.

Immediately after the infection the full-length genome accumulated and represented the dominant PCR product for all segments (Figure 3B). However, for the three polymerase genes the amount of full-length RNA decreased after 2 d p.i. while DI RNAs became clearly more abundant. Because shorter PCR products are preferentially amplified compared to larger products, the exact ratio of full-length to DI RNAs cannot be obtained from these results. However, the results are consistent with a general accumulation of DI RNAs soon after the infection of the virus bioreactor. Subsequently the amount of both full-length and DI segments declined at later stages of the cultivation experiment and the fluctuations found in virus titers (Figure 2B) also appeared for the PCR products (Figure 3B). More precisely, reductions in the amount of infectious virus titers coincide with a decrease of full-length polymerase genome segments and an increase of DI RNAs for segment 1–3. The accumulation of DIPs does not affect the HA titers immediately. However, without complete helper viruses DIPs are unable to replicate and virus particles are diluted in a continuous cultivation, leading to the observed time-shifted reduction of HA titers compared to infectious viruses (Figure 2B). For the same reason, such a delayed decline can also be found for segment 5 compared to full-length polymerase segments (Figure 3B), because accumulating DIPs still contain a full-length NP gene. Even though we used a non-quantitative PCR, the results are well in line with virus titer fluctuations and PCR results represent the average signal from the total virus particle population (infectious and non-infectious) which probably comprise diverse combinations of full-length and DI genome segments within single particles. The dilution of both standard viruses and DIPs leads to low MOIs within the virus bioreactor (Figure 2C). Under these conditions cells become infected by standard viruses without a co-infection by DIPs so that standard viruses accumulate again. With increasing amounts of helper viruses, DIPs can replicate and accumulate again to re-initiate the next phase.

To further analyze the process dynamics and to provide additional evidence that DIPs can cause the observed fluctuations in virus titers; we developed a mathematical model of our continuous virus infection system. In the past, similar models were successful in showing that cyclic variations can occur in serial passage infections due to the presence of defective interfering viruses [13], [20]. However, continuous cultivations require tailored modeling approaches which account for the constant dilution of reactor contents. In principle, this dilution may introduce oscillations itself which are independent of DIPs.

**Figure 4:**
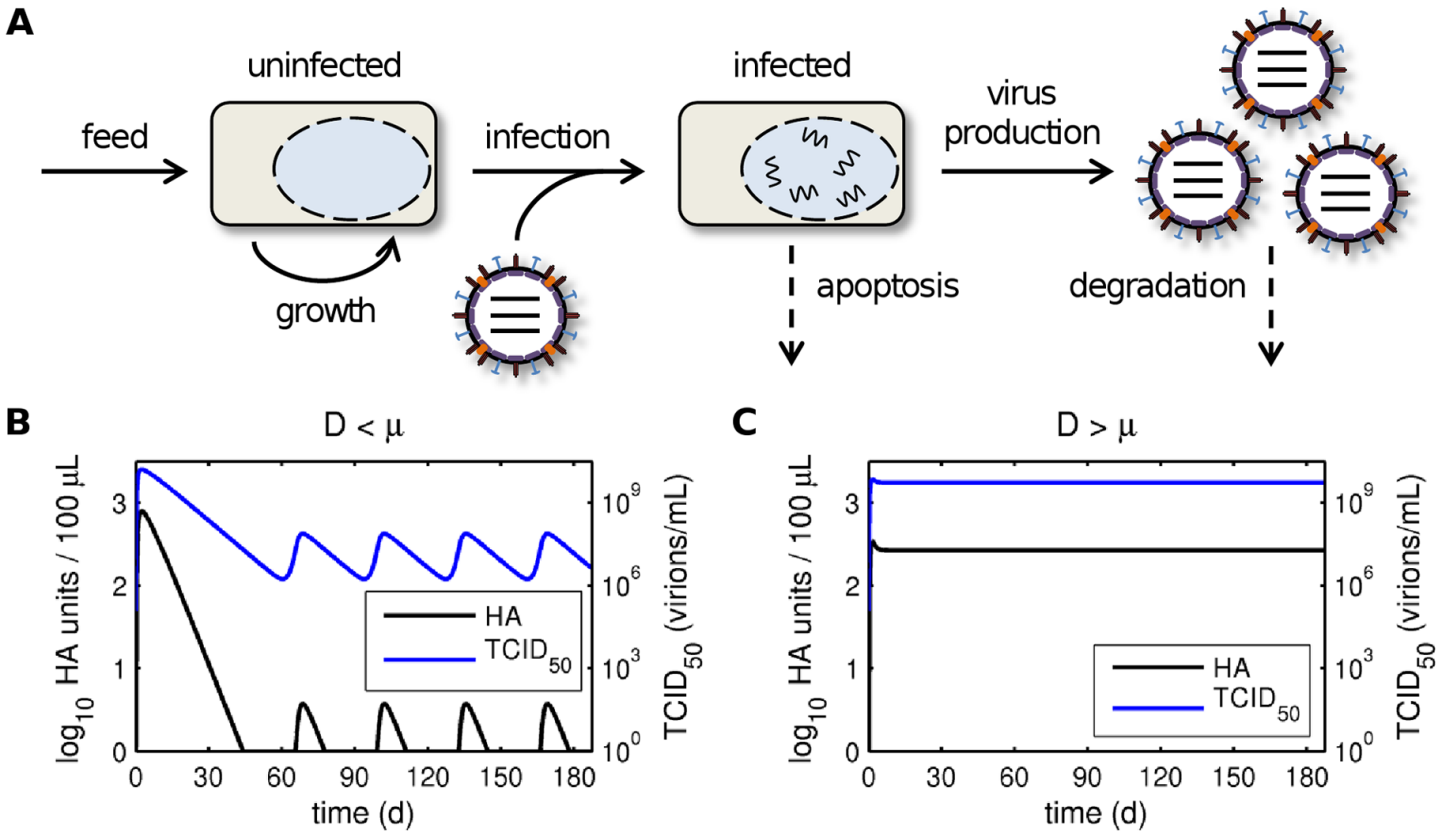
Model of continuous infection in the absence of DIPs. (A) Schematic representation of the model for continuous influenza A virus infection in the absence of DIPs (see Eq. (2)). The continuous harvest of cells and viruses was omitted for illustrative reasons. (B, C) Simulated virus titers for a dilution rate of the virus reactor D which is (B) lower than the specific growth rate µ and (C) higher than the specific growth rate µ. Parameters were chosen according to Table S1 except that the dilution rate in (B) was reduced to D = 10^−8^ 1/h.

To test this hypothesis, we first analyzed a simple model of continuous influenza A virus infection in the absence of DIPs (Figure 4A). The model focuses on the virus reactor and accounts for the continuous feed of uninfected cells, their exponential growth and infection, the production of standard virions, virus-induced apoptosis, degradation of free virus particles and the dilution of cells and virions. Mathematical analysis of this system indeed revealed parameter regions where a Hopf bifurcation gives rise to periodic solutions in the absence of DIPs (see section 3 in Text S1). In particular, oscillations can occur if and only if the dilution rate D of the virus reactor is lower than the specific growth rate µ of cells (Figure 4B). However, the time scale on which oscillations occur due to process mode is larger than observed in our experiments. More importantly, the dilution rate of the virus reactor in our setup (Figure 1) was higher than the maximum cell growth rate. According to the model, virus titers in our process should, thus, stay constant if the influence of DIPs can be neglected (Figure 4C).

**Figure 5:**
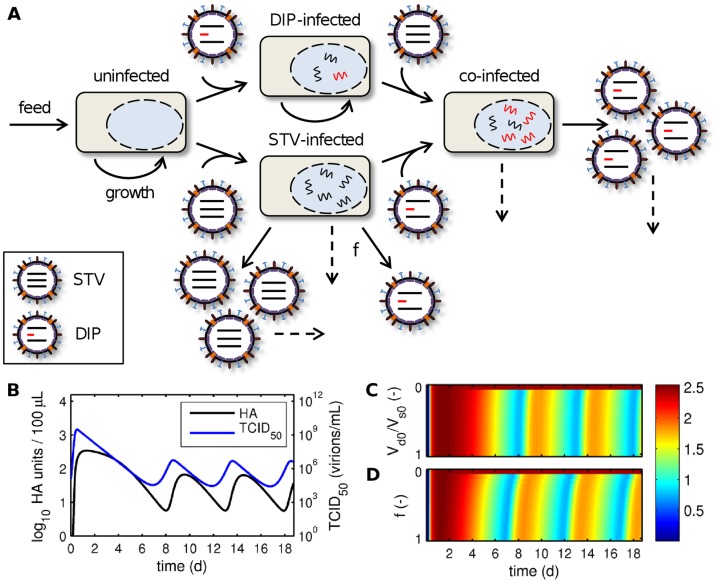
Model of continuous infection in the presence of DIPs. (A) Schematic representation of the model for continuous influenza A virus infection in the presence of DIPs (see Eq. (1)). Dashed arrows indicate apoptosis or virus degradation. The continuous harvest of cells and viruses was omitted for illustrative reasons. (B) Simulated virus titers for the parameters used in Table S1. (C, D) Log_10_ HA units/100 µL over process time for (C) various ratios of initial DIPs (V_d0_) to STVs (V_s0_) neglecting *de novo* DIP generation (f = 0) and (D) different rates of *de novo* DIP generation by STV-infected cells (f denoting the fraction of DIP to STV production) without DIPs being initially present (V_d0_ = 0).

Next, we introduced DIPs into our model to investigate how they may affect virus titers (Figure 5A). DIPs were simulated by defining a second, defective virus population which cannot replicate in cells in the absence of standard virus (STV). However, in cells co-infected with DIPs and STVs, replication proceeds leading to the production of DIPs. Furthermore, we considered the *de novo* generation of DIPs by cells infected with STV alone, which in addition to producing large quantities of STVs release a small amount of DIPs. Note that we do not distinguish between particles with defects in different segments but rather consider a general effect of an average DIP on virus production. Intriguingly, this simple model readily showed periodic oscillations even for D>µ and was able to reproduce the frequency observed in our experiments (Figure 5B). Other qualitative features of viral dynamics were also captured surprisingly well. For instance, the TCID_50_ in measurements and simulations starts with a large peak that drops to lower amplitudes later on. In addition, the number of infectious viruses decreases earlier than the HA titer. These observations confirm that DIPs are most likely causing the fluctuations in our continuous infection process. Note that in contrast to models for serial passage infections, which show chaotic fluctuations [13], [20], [21], our simulations yield regular oscillations with constant amplitude and frequency. Similar observations were made with a model for continuous baculovirus infection although the authors did not check whether their fluctuations originated from DIPs or are related to the process mode [22]. Overall, these regular patterns correspond well to our measurements suggesting a general characteristic of continuous infection processes. Hence, continuous systems lend themselves well to the analysis of DIP replication whereas serial passaging infections can become unpredictable [13]. The latter might be related to stochastic variations in the initial conditions of each passage [21].

Since fluctuations in HA titer reduce process yields, we used our model to test two approaches to avoid DIP formation: (i) minimizing the DIP concentration in the seed virus, e.g. by serial low-MOI passaging (Figure 5C), and (ii) reducing the extent of *de novo* DIP generation by STV-infected cells, e.g. by using optimized cell lines or virus strains (Figure 5D). In both cases fluctuations continued to emerge even with very pure seed viruses or low *de novo* DIP generation. Only when DIPs were completely removed from the seed and *de novo* DIP generation was eliminated simulations reached a steady state (see also Figure 4C). This observation was confirmed by mathematical analysis showing that the DIP-free regime is unstable upon the introduction of defective interfering viruses (see section 4 in Text S1). Hence, process optimization via the two tested strategies is unlikely to prevent oscillations and increase virus yield. Nevertheless, decreasing *de novo* DIP generation can slightly delay the first decrease in HA levels whereas titer fluctuations are surprisingly robust against changes in seed virus purity (compare Figure 5C and D). The latter was also observed in a model for serial passaging of VSV where the initial amount of DIPs had no effect on steady state virus titers [21]. Hence, given the right conditions DIPs can rapidly accumulate even from low levels posing a serious challenge not only to the continuous production of influenza vaccines but also to processes involving other viruses.

To our knowledge, this was the first time that a continuous production process for influenza viruses was established. Our data indicate, however, that continuous influenza virus propagation is compromised by the presence of DIPs. Indeed, DIPs accumulated shortly after infection when high MOI conditions were present within the virus bioreactor. Subsequently, infectious virus titers decreased dramatically followed by a delayed and less pronounced decrease of HA titers. These dynamics of infectious and non-infectious titers as well as their time-shifted recovery under low MOI conditions were observed similarly in the earliest studies about DIPs (formerly known as incomplete viruses) using undiluted serial passaging in embryonated eggs [16]. In line with the results presented in this study, a continuous propagation of baculoviruses using a cascade of two bioreactors also suffered from a decline of productivity [23], [24] which was caused by the accumulation of DIPs [25].

A continuous production process for rubella virus was successfully established [26], [27]. This virus is also capable of DIP formation [28] but has furthermore the ability to establish a persistent infection where virions are released without induction of a cytopathic effect. This property allows continuous virus propagation within a single bioreactor and may have contributed to the more uniform yields [26], [27]. Thus, continuous processes might only lead to constant virus titers for chronically infected cells or in the complete absence of DIPs. The generation of DIPs is a common phenomenon for different viruses [29] that may be difficult to control. However, the lack of lentiviral DIPs has been reported [30] and host cell factors have been described to be involved in the *de novo* generation or replication of DI genomes [31]–[33]. Consequently, further research is needed to elucidate which viral and host cell properties would enable the establishment of continuous virus production processes.

Our simple mathematical model of DIP replication during continuous infection captured the qualitative features of the measurements surprisingly well. Nevertheless, a comprehensive understanding of influenza virus infection may require further modeling work. For instance, Thompson *et al.* suggested that different populations of co-infected cells exist, which may produce a mixture of standard and defective viruses [34]. Furthermore, they emphasized the need to mechanistically understand how defective genomes interfere with intracellular replication. Models of DIP growth during influenza virus infection in general and our approach in particular may, hence, benefit from a quantitative description of intracellular virus replication [35]. Such detailed models could help to increase yields in continuous vaccine production by suggesting new strategies to suppress DIP replication at the intracellular level.

## Conclusions

Influenza A virus can be propagated in continuous culture using the robust and fast-growing suspension cell line AGE1.CR. However, a stable long-term virus production with constant high titers is impeded by defective interfering particles. Our experimental data as well as our modeling approach demonstrate that DIPs rapidly accumulate during continuous virus propagation and, thus, represent a severe challenge for the productivity of the system. Since virus titers during continuous infection show regular oscillations, as opposed to chaotic fluctuations during serial passaging, continuous systems lend themselves well to the investigation of *in vitro* DIP replication. Additionally, the continuous influenza virus cultivation using a two-stage bioreactor setup can serve as a novel tool to study aspects of viral evolution.

## Supporting Information

Table S1 **Parameters and non-zero initial conditions used for numerical simulations.**
(DOCX)

Text S1 **Theoretical analysis for the models (1) and (2).**
(DOCX)
