# Supplementary material for: Continuous Influenza Virus Production in Cell Culture Shows a Periodic Accumulation of Defective Interfering Particles
Source: PLoS One. 2013 Sep 5;8(9):e72288. doi: 10.1371/journal.pone.0072288 (PMC3764112; doi:10.1371/journal.pone.0072288)
Supplement: Table S1 — Parameters and non-zero initial conditions used for numerical simulations. (DOCX) [file pone.0072288.s001.docx]

**Table S1:** Parameters and non-zero initial conditions used for numerical simulations.

| Symbol | Description | Value | Unit |
| --- | --- | --- | --- |
| $\mu$ | specific growth rate | $0.027$ | $1/\text{h}$ |
| $D$ | dilution rate of virus reactor | $0.0396$ | $1/\text{h}$ |
| $f$ | fraction of produced DIPs | $10^{-3}$ | - |
| $k_{1}$ | virus infection rate | $2.12 \text{x} 10^{-9}$ | $\text{mL}/\left(\text{virion}\cdot\text{h}\right)$ |
| $k_{2}$ | apoptosis rate of infected cells | $7.13 \text{x} 10^{-3}$ | $1/\text{h}$ |
| $k_{3}$ | virus production rate | $168$ | $\text{virions}/\left(\text{cell}\cdot\text{h}\right)$ |
| $k_{4}$ | virus degradation rate | $0.035$ | $1/\text{h}$ |
| $T_{0}$ | initial target cell concentration | $5 \text{x} 10^{6}$ | $\text{cells}/\text{mL}$ |
| $T_{in}$ | cell concentration in the feed | $3 \text{x} 10^{6}$ | $\text{cells}/\text{mL}$ |
| $V_{s0}$ | initial STV concentration | $1.25 \text{x} 10^{5}$ | $\text{virions}/\text{mL}$ |
