# Supplementary material for: Continuous Influenza Virus Production in Cell Culture Shows a Periodic Accumulation of Defective Interfering Particles
Source: PLoS One. 2013 Sep 5;8(9):e72288. doi: 10.1371/journal.pone.0072288 (PMC3764112; doi:10.1371/journal.pone.0072288)
Supplement: Text S1 — Theoretical analysis for the models (1) and (2). (DOCX) [file pone.0072288.s002.docx]

**Continuous Influenza Virus Production in Cell Culture Shows a Periodic Accumulation of Defective Interfering Particles**

**Timo Frensing, Frank Stefan Heldt, Antje Pflugmacher, Ilona Behrendt, Ingo Jordan, Dietrich Flockerzi, Yvonne Genzel, Udo Reichl**

**Text S1: Theoretical analysis for the models (1) and (2)**

1. **Scaled DIP-Model in 6D**

We consider the 6D system (1) with the 8 positive parameters $D$, $T_{in}$, $\mu$, $k_{1}$, $k_{2}$, $k_{3}$, $k_{33}$, $k_{4}$, and $f\geq0$ and are mostly interested in nonnegative initial values of the form $T\left(0\right)=T_{0}\geq0$, $I_{d}\left(0\right)=I_{s}\left(0\right)=I_{c}\left(0\right)=0$, $V_{s}\left(0\right)=V_{s0}\geq0$ and $V_{d}\left(0\right)=V_{d0}\geq0$. We investigate the case with $f=0$. A small positive $f$ won’t change much the dynamics in but we could always take $V_{d}\left(0\right)$ to be $0$. Note that the nonnegative orthants and the one with $I_{d}=I_{c}=V_{d}=0$ are positive invariant.

We will provide a structural analysis of system (1) for arbitrary parameter values. In particular, we will prove first that the structure of system (1) without DIPs $\left(I_{d}=I_{c}=V_{d}=0\right)$ excludes a Hopf bifurcation to periodic solutions for $D>\mu$. In a second step, we show that, in contrast, system (1) with DIPs allows a Hopf bifurcation to periodic oscillations for $D>\mu$. To this end we reduce the number of parameters by introducing the scalings

| $\tau=\left(k_{2}+D\right)t$ and $\xi=\frac{k_{1}}{k_{2}+D}col\left(T, I_{s},V_{s}\right)$, $\eta=\frac{k_{1}}{k_{2}+D}col\left(I_{d}, I_{c}, V_{d}\right)$ | (S1.1) |
| --- | --- |

with $\xi=col\left(\xi_{1},\xi_{2},\xi_{3}\right)$, $\eta=col\left(\eta_{1},\eta_{2},\eta_{3}\right)$ and arrive at the 6D-system

| $\frac{d\xi_{1}}{d\tau}=u+\left(\nu-\xi_{3}\right)\xi_{1}-\xi_{1}\eta_{3},$ | $\xi_{1}\left(0\right)=\frac{k_{1}T_{0}}{k_{2}+D},$ | (S1.2a) |
| --- | --- | --- |
| $\frac{d\xi_{2}}{d\tau}=\xi_{1}\xi_{3}-\xi_{2}-\xi_{2}\eta_{3},$ | $\xi_{2}\left(0\right)=0,$ | (S1.2b) |
| $\frac{d\xi_{3}}{d\tau}=\alpha\xi_{2}-\left(\xi_{1}+\xi_{2}+\eta_{1}+\eta_{2}+\beta\right)\xi_{3},$ | $\xi_{3}\left(0\right)=\frac{k_{1}V_{s0}}{k_{2}+D},$ | (S1.2c) |
| $\frac{d\eta_{1}}{d\tau}=\left(\nu-\xi_{3}\right)\eta_{1}+\xi_{1}\eta_{3},$ | $\eta_{1}\left(0\right)=0,$ | (S1.2d) |
| $\frac{d\eta_{2}}{d\tau}=\xi_{3}\eta_{1}-\eta_{2}+\xi_{2}\eta_{3},$ | $\eta_{2}\left(0\right)=0,$ | (S1.2e) |
| $\frac{d\eta_{3}}{d\tau}=\alpha_{3}\eta_{2}-\left(\xi_{1}+\xi_{2}+\eta_{1}+\eta_{2}+\beta\right)\eta_{3},$ | $\eta_{3}\left(0\right)=\frac{k_{1}V_{d0}}{k_{2}+D},$ | (S1.2f) |

with the 5 parameters

| $u=\frac{k_{1}DT_{in}}{\left(k_{2}+D\right)^{2}}>0,$ | | $\nu=\frac{\mu-D}{k_{2}+D}<0,$ | | (S1.3a) |
| --- | --- | --- | --- | --- |
| $\alpha=\frac{k_{3}}{k_{2}+D}>0,$ | $\alpha_{3}=\frac{k_{33}}{k_{2}+D}>0,$ | | $\beta=\frac{k_{4}+D}{k_{2}+D}>0.$ | (S1.3b) |

In the new variables, we obtain a system of the form

|  | $\xi\left(0\right)=\xi_{0}$, | (S1.4a) |
| --- | --- | --- |
|  | $\eta\left(0\right)= \eta_{0}$, | (S1.4b) |

with $U=\left(u,0,0\right)^{T}$ and

| $A\left(\xi\right)=\left(\begin{matrix} -\left(\left\vert\nu\right\vert+\xi_{3}\right) & 0 & 0 \\ \xi_{3} & -1 & 0 \\ 0 & \alpha& -\left(\xi_{1}+\xi_{2}+\beta\right) \end{matrix}\right),$ | $B\left(\xi\right)=\left(\begin{matrix} 0 & 0 & \xi_{1} \\ 0 & 0 & \xi_{2} \\ \xi_{3} & \xi_{3} & 0 \end{matrix}\right),$ | (S1.4c) |
| --- | --- | --- |
| $C\left(\xi,\eta\right)=\left(\begin{matrix} -\left(\left\vert\nu\right\vert+\xi_{3}\right) & 0 & \xi_{1} \\ \xi_{3} & -1 & \xi_{2} \\ 0 & \alpha_{3} & -\left(\xi_{1}+\xi_{2}+\eta_{1}+\eta_{2}+\beta\right) \end{matrix}\right).$ | | (S1.4d) |

The octant is positive invariant with the DIP-free reduced system

|  | $\xi\left(0\right)=\xi_{0}.$ | (S1.5) |
| --- | --- | --- |

Moreover, the octant $\left\{\xi_{1}\geq0, \xi_{2}=\xi_{3}=0, \eta_{j}\geq0\right\}$ and the positive $\xi_{1}$-axis are positive invariant. We observe in (S1.3) that the new parameters depend linearly on $T_{in}$, $k_{3}$, $k_{4}$, $k_{33}$ and $\mu$ whereas the dependence on $D$ is rather involved.

1. **DIP-free Model in 3D**

To test whether the oscillations in virus titers are caused by DIPs or the continuous process mode, we use the reduced model for $\xi$ neglecting defective particles from
$\eta$. In this 3D case, we prefer the notation $\left(x,y,z\right)^{T}$ instead of $\left(\xi_{1},\xi_{2},\xi_{3}\right)^{T}$ so that system (S1.5) reads

| $\frac{dx}{d\tau}=u+\left(\nu-z\right)x,$ | $x\left(0\right)=x_{0}=\frac{k_{1}T_{0}}{k_{2}+D},$ | (S2.1a) |
| --- | --- | --- |
| $\frac{dy}{d\tau}=xz-y,$ | $y\left(0\right)=0,$ | (S2.1b) |
| $\frac{dz}{d\tau}=\alpha y-\left(x+y+\beta\right)z,$ | $z\left(0\right)=z_{0}=\frac{k_{1}V_{s0}}{k_{2}+D},$ | (S2.1c) |

with the 4 parameters $u$, $\nu$, $\alpha$ and $\beta$ from (S1.3). The sign of $\nu$ is not fixed. The positive x-axis is positive invariant. For $\nu<0$ one has the globally exponentially stable equilibrium $x=-u/\nu$ on it.

We turn to the *nonnegative equilibria* $E=col\left(x,y,z\right)$ of (S2.1) on the boundary of
. We first note that $x$ cannot be $0$ because of $u>0$ (cf. (S2.1a)). In case $z=0=y$ (cf. (S2.1b)) one has $x=-u/\nu>0$ for $\nu<0$. There's no nonnegative equilibrium of the form $E=col\left(x,0,0\right)$ for $\nu\geq0$.

We now come to the *positive equilibria* $E=col\left(x,y,z\right)$ of (S2.1). The equation can be rewritten as

| $\nu=z-\frac{u}{x},$ | (S2.2a) |
| --- | --- |

the equation can be rewritten as

| $x+xz+\beta=\alpha x,$ | i.e., | $\beta=\left(\alpha-1-z\right)x$, | (S2.2b) |
| --- | --- | --- | --- |

The equation

| $y=xz,$ | (S2.2c) |
| --- | --- |

originating from , and the relations (S2.2a) and (S2.2b) can be seen as defining equations for positive steady states parameterized by

| $x>0, z\in\left(0, \alpha-1\right), u>0$ | and | $\alpha>1.$ | (S2.2d) |
| --- | --- | --- | --- |

**Lemma 2.1** *Just in case* $\nu<0$*, system (S2.1) allows a nonnegative equilibrium on the boundary of* *, namely* $E_{0}=col\left(-\frac{u}{\nu}, 0, 0\right)$*. System (S2.1) possesses a unique positive equilibrium* $E_{1}=col\left(x,y,z\right)$ *with*

| $0<z=\frac{\left(\alpha-1\right)u+\beta\nu}{u+\beta}\in\left(\nu, \alpha-1\right),$ | $x=\frac{u+\beta}{\alpha-1-\nu},$ | $y=xz,$ | (S2.3a) |
| --- | --- | --- | --- |

*where the side conditions can be rewritten in terms of the parameters as*

| $\alpha>1 \text{and} \alpha-1>\nu>-\frac{\left(\alpha-1\right)u}{\beta}:=\nu_{min}.$ | (S2.3b) |
| --- | --- |

1. **Bifurcation Analysis in 3D**

The Jacobian of (S2.1) at a nonnegative equilibrium $E$ is given by

| $Jac\left(E\right)=\left[\begin{matrix} \nu-z & 0 & -x \\ z & -1 & x \\ -z & \alpha-z & -\left(x+y+\beta\right) \end{matrix}\right]$ | (S3.1b) |
| --- | --- |

in terms of 6 variables which are related by the equilibrium conditions in (S2.2).

**Lemma 3.1** *The Jacobian* $J_{0}:=Jac\left(E_{0}\right)$ *at the boundary equilibrium* $E_{0}$ *possesses three negative eigenvalues for* $\nu<\nu_{min}$ *and exactly two negative eigenvalues for* $\nu\in\left(\nu_{min},0\right)$*. A transcritical stationary bifurcation gives rise to the positive equilibrium* $E_{1}$ *for* $\nu>\nu_{min}$*. The Jacobian* $J_{+}:=Jac\left(E_{1}\right)$ *at the positive equilibrium* $E_{1}$ *possesses three negative eigenvalues for* $\nu$ *close to* $\nu_{min}$*. For negative* $\nu$*'s, the real parts of the eigenvalues of* $J_{+}$ *stay negative, so that there does not occur a Hopf bifurcation to periodic solutions in the nonlinear system (S2.1) at* $E_{1}$ *for negative* $\nu$*'s.*

*Proof:*

1. For negative $\nu$, one has

| $J_{0}:=Jac\left(E\right)=\left[\begin{matrix} \nu& 0 & -u/\nu\\ 0 & -1 & u/\nu\\ 0 & \alpha& u/\nu-\beta\end{matrix}\right].$ | (S3.2) |
| --- | --- |

It has the negative eigenvalue $\nu$ with the x-axis as eigenspace. The $\left(2\times2\right)$-block possesses the negative trace $-1-\beta-u/\left|\nu\right|$ and the determinant $\beta+\left(\alpha-1\right)u/\left|\nu\right|$. Thus one encounters the eigenvalue $0$ exactly for $\nu=\nu_{min}$. A transcritical stationary bifurcation happens giving rise to the positive equilibrium: For $\nu$'s with (S2.3b) there exists the positive equilibrium $E_{1}$ emanating at $\nu=\nu_{min}$from $E_{0}$ (which exists for all negative $\nu$'s).

1. At positive equilibria $E_{1}=col\left(x,y,z\right),$ the Jacobian (S3.1) can be rewritten as

| $J_{+}:=Jac\left(E_{1}\right)=\left[\begin{matrix} -u/x & 0 & -x \\ z & -1 & x \\ -z & \alpha-z & -\alpha x \end{matrix}\right]=\left[\begin{matrix} \nu-z & 0 & -x \\ z & -1 & x \\ -z & \alpha-z & -\alpha x \end{matrix}\right]$ | (S3.3) |
| --- | --- |

in terms of 5 variables. We seek conditions for pure imaginary eigenvalues of $J_{+}$. So we investigate the characteristic polynomial

| $\chi\left(\lambda\right):=\det\left(\lambda\text{I}-\text{J}_{+}\right)=\lambda^{3}+\lambda^{2}A_{2}+\lambda A_{1}+A_{0}$ | (S3.4a) |
| --- | --- |

with positive coefficients

| $A_{2}=\left(z-\nu\right)+\alpha x+1, A_{1}=\left(z-\nu\right)\left(\alpha x+1\right), A_{0}=xz\left(\alpha-1-z\right)$. | (S3.4b) |
| --- | --- |

Thus the Jacobian $J_{+}$ does not possess a nonnegative real eigenvalue and it has the negative determinant $-A_{0}$. Hence, for sufficiently small positive $x$, $J_{+}$ has 3 negative eigenvalues. We investigate whether these eigenvalues can cross the imaginary axis at some nonzero points $\pm i\omega$. In order to have a nonzero pure imaginary eigenvalue $i\omega$ one has to solve the necessary and sufficient conditions

| $0\mathop{=}\limits^{!} Re(\chi\left(i\omega\right), 0\mathop{=}\limits^{!} Im(\chi\left(i\omega\right).$ |  |
| --- | --- |

This amounts to solving $\omega^{2}=A_{1}=A_{0}/A_{2}$, i.e.,

| $\left(\frac{u}{x}+\alpha x+1\right)\frac{u}{x} \left(\alpha x+1\right)=\left(z-\nu+\alpha x+1\right)\left(\left(z-\nu\right)\left(\alpha x+1\right)\right)=xz\left(\alpha-1-z\right)$. | (S3.5) |
| --- | --- |

Here, we consider the variables $u$, $\alpha$, $x$ and $z$ from (S2.2d) as parameters defining an equilibrium via the equations (S2.2a)-(S2.2c).

1. We show that, given an admissible solution $z=Z_{1/2}\left(x,u,a\right)$ of the quadratic equation (S3.5) (wrt. $z$), the corresponding $\nu=z-u/x$ from (S2.2a) is necessarily nonnegative. To this end, we scale $z$ via $z=\rho\frac{u}{x}$ introducing the new positive variable $\rho$ so that equation (S3.5) is equivalent to

| $q\left(u,\alpha,x,\rho\right):=\left(u+x\left(\alpha x+1\right)\right)\left(\alpha x+1\right)-\rho x\left(\left(\alpha-1\right)x-\rho u\right)=0$. | (S3.6) |
| --- | --- |

Because of

| $q\left(u,\alpha,x,\rho\right)\geq q\left(0,\alpha,x,\rho\right)=x\left(\alpha^{2}x^{2}+\left(2-\rho\right)\alpha x+1+\rho x\right)$ | (S3.7) |
| --- | --- |

a necessary condition for having an admissible solution of (S3.5) is $\rho>2$. Hence, equation (S3.5) does not have an admissible solution for which $\nu$ is negative $\left(\rho\in\left(0,1\right)\right)$.

**Remark 3.2 (Hopf bifurcation to periodic oscillations)**

The right-hand side of (S3.5) represents a $u$-independent quadratic polynomial in $z$which is positive on $\left(0, \alpha-1\right)$ with maximal value $x\left(\frac{\alpha-1}{2}\right)^{2}$. The left-hand side of (S3.5) is independent of $z$ and is tending to $0$ for $u\to0$. Thus, for sufficiently small $u$, one has explicit formulae for the two zeros $z=Z_{1/2}\left(x,u,\alpha\right)$ of (S3.5): So given $x>0$, $\alpha>1$ and a sufficiently small $u>0$ one first has solutions $z=Z_{1/2}\left(x,u,\alpha\right)$ and then $y=xz>0$, $\nu=z-\frac{u}{x}$ and $\beta=\left(\alpha-1-z\right)x>0$ for the equilibrium values (cf. (S2.2)). The condition $\rho>2$ from (S3.7) can be written as

| $\nu=\rho\frac{u}{x}-\frac{u}{x}\mathop{>}\limits^{!} \frac{u}{x}=\frac{u}{u+\beta}\left(\alpha-1-\nu\right)>0.$ |  |
| --- | --- |

So, recalling (S2.3a), we arrive at the necessary conditions

| $\alpha>1 \text{and} \alpha-1>\nu>\left(\alpha-1\right)\frac{1}{2+\beta/u }$ | (S3.8) |
| --- | --- |

asking for a sufficiently small $u$ in case of $0<\nu<\left(\alpha-1\right)/2$. In the original parameters, (S3.8) is given by

| $D+\frac{\left(k_{3}-k_{2}-D\right)Dk_{1}T_{in}}{\left(k_{2}+D\right)\left(k_{4}+D\right)+2k_{1}DT_{in}}<\mu<k_{3}-k_{2}$ | (S3.9) |
| --- | --- |

asking in particular for $D<\mu$ and $k_{2}+D<k_{3}$.

There are explicit - simple but tedious - formulae for sufficient conditions. The period near the bifurcation point is $2\pi/\omega$ in first approximation. The amplitude of the oscillation near the bifurcation point has an explicit approximation formula [[1](#_ENREF_1)]. For a numerical example one might take

| $x=1, \alpha=2, u=\frac{1}{6}\left(-9+\sqrt{82.08}\right), z=0.1, y=0.1, \nu=0.1-u, \beta=0.9$ | (S3.10) |
| --- | --- |

and vary $\alpha$ near $2$ or $u$ near $0.01$.

1. **Periodic oscillations in the 6D DIP-model**

We investigate the 6D system (S1.2) in case of $\nu<0$, $f=0$ and $V_{d}\left(0\right)>0$. We turn to the determination of positive equilibria $E_{2}$ of the 6-dimensional system (S1.2) and parameterize the equilibrium values by $\xi_{1}$, $\xi_{3}$, $u$, $\alpha$ and $\eta_{3}$. With $\gamma:=\left|\nu\right|+\xi_{3}$, the equations (S1.2a), (S1.2b) and (S1.2d) provide

| $\left\vert v\right\vert=\frac{u}{\xi_{1}}-2\eta_{3}$, and $\gamma=\frac{u}{\xi_{1}}-\eta_{3}$, $\xi_{2}=\frac{\xi_{1}\xi_{3}}{1+\eta_{3}}$, $\eta_{1}=\frac{\xi_{1}\eta_{3}}{\gamma}$. | (S4.1a) |
| --- | --- |

The remaining equations (S1.2c), (S1.2e) and (S1.2f) then lead to

| $\eta_{2}=\left(\frac{\xi_{1}\xi_{3}}{\gamma}+\xi_{2}\right)\eta_{3}, \beta=\frac{\alpha\xi_{2}}{\xi_{3}}-\left(\xi_{1}+\xi_{2}+\eta_{1}+\eta_{2}\right),$ | (S4.1b) |
| --- | --- |

and finally to

| $\alpha_{3}=\frac{\eta_{3}}{\eta_{2}}\frac{\alpha\xi_{2}}{\xi_{3} }=\frac{\alpha\gamma}{\xi_{3}\left(1+\eta_{3}+\gamma\right)}$ | (S4.1c) |
| --- | --- |

reducing on $\eta=0$ to

| $\alpha_{3}=\alpha_{3}^{c}:=\frac{\alpha u}{\xi_{3}\left(\xi_{1}+u\right)}.$ | (S4.2) |
| --- | --- |

It is a tedious task to derive the conditions that guarantee the realizability of these parameter values in terms of the original parameters in system (1). We note that the determinant of $C\left(\xi,\eta\right)$ vanishes along (S4.1) for . Moreover, taking $\eta_{3}\to0_{+}$ in (S4.1), the positive equilibrium tends to the equilibrium . This limiting equilibrium $E_{1}\times\left\{0\right\}$ is critical in the sense that its Jacobian possesses $0$ as an eigenvalue (giving rise to the bifurcating $E_{2}$).

The over-all stability in of $E_{1}$ is determined by the eigenvalues of the Jacobian of (S1.4a) which is in block-triangular form

|  | (S4.3) |
| --- | --- |

where we have suppressed the remaining parameters. The eigenvalues of the upper left block $F_{\xi}\left(\xi\right)$ are in the left half-plane of by Lemma 3.1 since $\nu$ has been assumed to be negative. We note that the matrix $C\left(\xi,0\right)$ in (S4.3) is an exponentially stable matrix exactly for $\alpha_{3}<\alpha_{3}^{c}$ at any boundary equilibrium $E_{1}\times\left\{0\right\}=\left(\xi_{1},\xi_{2},\xi_{3}\right)^{T}\times\left\{0\right\}$ [[2](#_ENREF_2)]. First, because of the positive invariance of , $C\left(\xi,0\right)$ cannot possess non-real eigenvalues. Secondly, considering $\alpha_{3}=\alpha_{3}^{c}+\delta$, there is exactly one eigenvalue of $C\left(\xi,0\right)$ passing transversally from the left to the right half-plane in when $\delta$ passes from negative to positive values. This follows easily from the representation

| $\det\left(\lambda I-C\left(\xi,0\right)\right)=\lambda^{3}+d_{2}\lambda^{2}+d_{1}\lambda+d_{0}$ | (S4.4a) |
| --- | --- |

of the characteristic polynomial with the coefficients

| $d_{2}=1+\gamma+\alpha\xi_{1}>0, d_{1}=\alpha\xi_{1}+\gamma+\alpha u-\alpha_{3}\xi_{1}\xi_{3}, d_{0}=\alpha u-\alpha_{3}\xi_{3}\left(u+\xi_{1}\right)$. | (S4.4b) |
| --- | --- |

The relations in (S4.4) show that $C\left(\xi,0\right)$ possesses the eigenvalue $\lambda_{0}=\lambda_{0}\left(\alpha_{3}\right)$ that is vanishing at $\alpha_{3}=\alpha_{3}^{c}$with a positive $\alpha_{3}^{c}$-derivative at $\alpha_{3}^{c}[\lambda_{0}^{'}\left(\alpha_{3}^{c}\right)>0$ because of $\alpha_{3}^{c}\xi_{1}\xi_{3}=\frac{\alpha u}{\gamma+1}<\alpha u]$. The values given by (S4.1) with $\eta=0$ thus provide a critical point in the space of parameters $\alpha_{3}^{c}$, $\alpha^{c}$, $u^{c}$, $\nu^{c}$, $\beta^{c}$ and states $\xi_{1}^{c}$, $\xi_{2}^{c}$, $\xi_{3}^{c}$which induces a transcritical steady-state bifurcation to the exponentially stable positive equilibrium $E_{2}$.

Varying, for example, $u$ away from criticality may entail a subsequent Hopf bifurcation from the positive equilibrium $E_{2}$ at some parameter value $u_{Hopf}$. Based on numerical simulations, this seems indeed to be the case.

# Supplementary references

1. Kuznet︠s︡ov IUA (1998) Elements of applied bifurcation theory. New York: Springer. 591 p.

2. Berman A, Plemmons RJ (1994) Nonnegative matrices in the mathematical sciences. Philadelphia: Society for Industrial and Applied Mathematics. 340 p.
